# Central Nervous System Whipple Disease Presenting as Hypersomnolence

**DOI:** 10.7759/cureus.23572

**Published:** 2022-03-28

**Authors:** Marcela A de Oliveira Santana, Saira Butt, Mehdi Nassiri

**Affiliations:** 1 National Reference Center for Sanitary Dermatology and Leprosy, Federal University of Uberlândia, Uberlândia, BRA; 2 Infectious Disease, Indiana University School of Medicine, Indianapolis, USA; 3 Pathology and Laboratory Medicine, Indiana University School of Medicine, Indianapolis, USA

**Keywords:** hypersomnolence, hydrocephalus, tropheryma whipplei, central nervous system, whipple disease

## Abstract

Whipple disease (WD) is a rare systemic infection caused by *Tropheryma whipplei (T. whipplei)*. Its clinical features are broad, and atypical clinical patterns such as the involvement of the heart, lungs, or the central nervous system (CNS) can occur. We report a case of a 58-year-old man who had been previously diagnosed with classic WD; he was evaluated for functional decline, extreme somnolence, and recurrent admissions for hydrocephalus. The patient was diagnosed with a neurologic relapse of WD after a positive *T. whipplei *polymerase chain reaction (PCR) from a cerebral spinal fluid (CSF) sample. He was successfully treated with IV ceftriaxone followed by oral trimethoprim-sulfamethoxazole (TMP-SMX). In classic WD, the CNS symptoms usually present in the late phase of the disease or in the form of relapse, especially after an inadequate treatment course. This case highlights the importance of considering CNS involvement in WD when a patient with a previous history of classic WD presents with hypersomnolence, hydrocephalus, or other neurologic symptoms.

## Introduction

Whipple disease (WD) is a rare systemic infection caused by *Tropheryma whipplei (T. whipplei)*, a slow-replicating rod-shaped Gram-positive actinomycete bacteria [1]. The hallmark of WD is the invasion of the intestinal mucosa with macrophages incompetent to degrade *T. whipplei*. Its clinical presentation is broad, ranging from acute transient disease, asymptomatic carriage, and localized extra-intestinal disease, to classic systemic disease and WD in association with immunosuppression [2]. Classic WD is characterized by diarrhea, abdominal pain, and weight loss and is generally seen in middle-aged men. Various other clinical patterns, such as the involvement of the heart, lungs, or central nervous system (CNS) can also occur. CNS WD can have three types of presentations: the classic disease with neurologic symptoms, relapse, or isolated neurologic disease [3]. CNS WD usually manifests as a memory disorder, personality change, or dementia. Other frequent clinical signs include ophthalmoplegia, nystagmus, or myoclonia in combination with disturbed sleep patterns, ataxia, seizure, or symptoms of cerebral compression due to hydrocephalus. CNS and ocular symptoms, such as blurred vision or ophthalmoplegia, may occur with minimal or no gastrointestinal involvement [4]. CNS WD can also present as chronic encephalitis. Besides WD, indolent onset is also common in some other causes of chronic encephalitis such as syphilis, subacute sclerosing panencephalitis (measles), Lyme disease, African trypanosomiasis, bartonellosis, autoimmune encephalopathy with anti-N-methyl-D-aspartate receptor antibody, HIV-1 infection, and progressive multifocal leukoencephalopathy. Although chronic encephalitis symptoms often overlap, dementia and personality change are the early and prominent signs of CNS WD [5].

The differential diagnosis is extensive for WD and a correct diagnosis can be delayed for years. The definitive diagnosis can be challenging as *T. whipplei* has a slow growth pattern and is difficult to isolate on culture due to the need for a living eukaryotic host cell [1]. Histopathology showing inclusions within macrophages staining with periodic acid-Schiff (PAS) is the classic diagnostic tool. This method has low specificity and sensitivity and often requires several samples from the duodenum mucosa due to the variable distribution of the disease. Lately, polymerase chain reaction (PCR) has been used, often in combination with biopsy, for diagnosis confirmation, and it is especially valuable to investigate samples from variable tissues or body fluids, such as the cerebral spinal fluid (CSF) [2]. The clinical picture, together with pathognomonic PAS-positive histology from the duodenum, may usually be sufficient to establish the diagnosis. A specific diagnostic test, such as PCR and/or immunohistochemistry, is recommended in every newly identified patient and is mandatory in cases of doubt or if the diagnosis is based on extra-duodenal tissue [1,3].

In this report, we present a case of a middle-aged man with functional decline, extreme somnolence, and recurrent admissions for hydrocephalus who was diagnosed with a neurologic relapse of WD.

## Case presentation

A 58-year-old man was brought to the hospital by his family due to progressive functional decline and hypersomnolence over the last two months. About 10 years ago, the patient had experienced bronzed skin, weight loss, arthralgias, and chronic diarrhea for three years. He had been diagnosed with WD at that time after a small bowel biopsy had revealed PAS-positive inclusions within macrophages. He had been prescribed trimethoprim-sulfamethoxazole (TMP-SMX), which he would take for a few months but subsequently stopped after the improvement in his condition, and he had been lost to follow-up.

**Figure 1:**
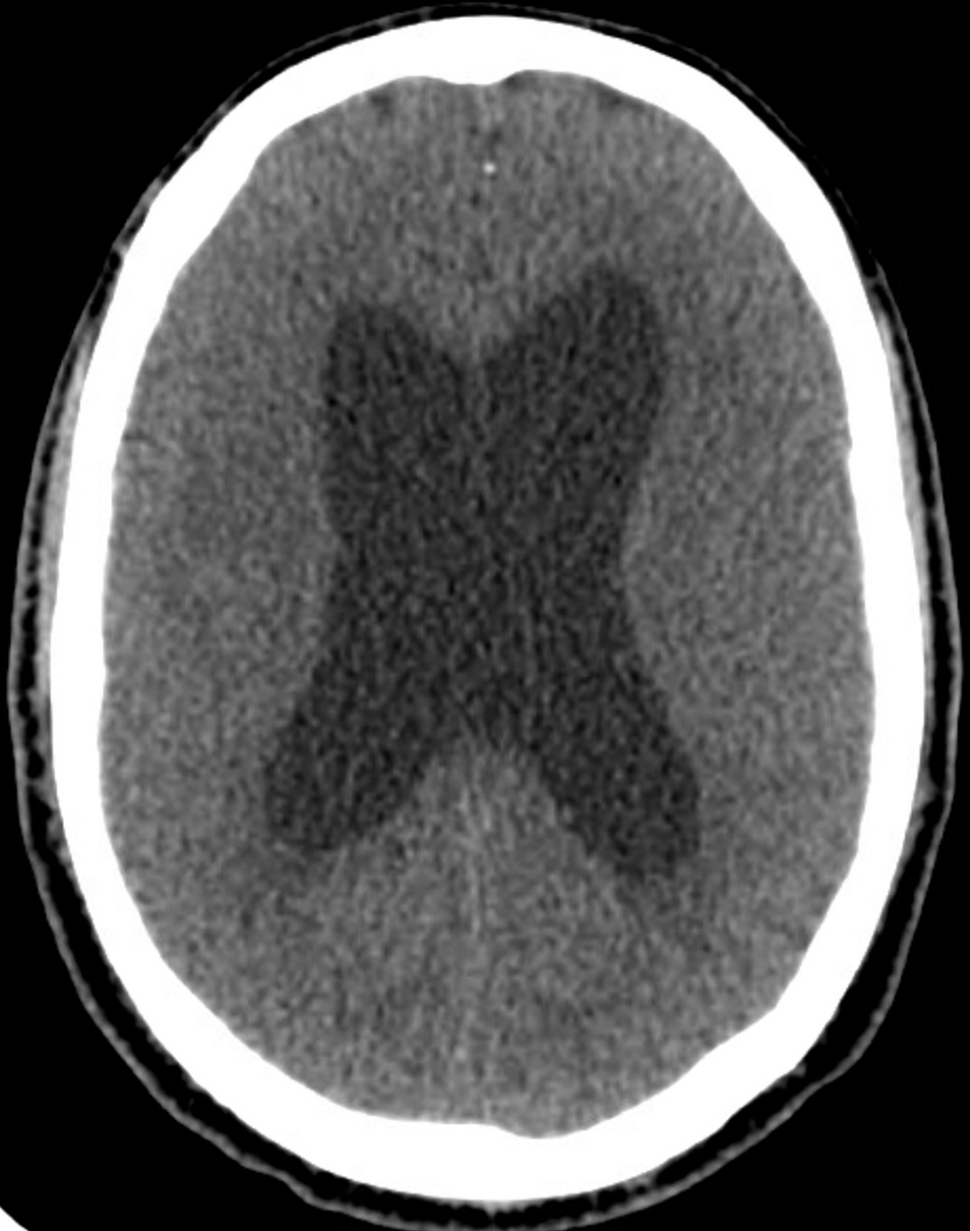
Brain CT showing ventriculomegaly CT: computed tomography

Two years prior to the current presentation, the patient had been admitted with psychomotor slowing, difficulty with speech, urinary incontinence, gait instability, and frequent falls. A brain CT had revealed ventriculomegaly at that time (Figure 1). After extensive workup, he had been diagnosed with idiopathic normal pressure hydrocephalus (NPH) and had undergone endoscopic third ventriculostomy (ETV). His condition had dramatically improved post-procedure and he had experienced a normal functional status for about eight months. However, after the initial improvement, he had recurrent admissions for progressive functional decline and had undergone a ventriculoperitoneal (VP) shunt placement, which had been adjusted periodically, but the patient had never returned to normal clinical function.

**Figure 2:**
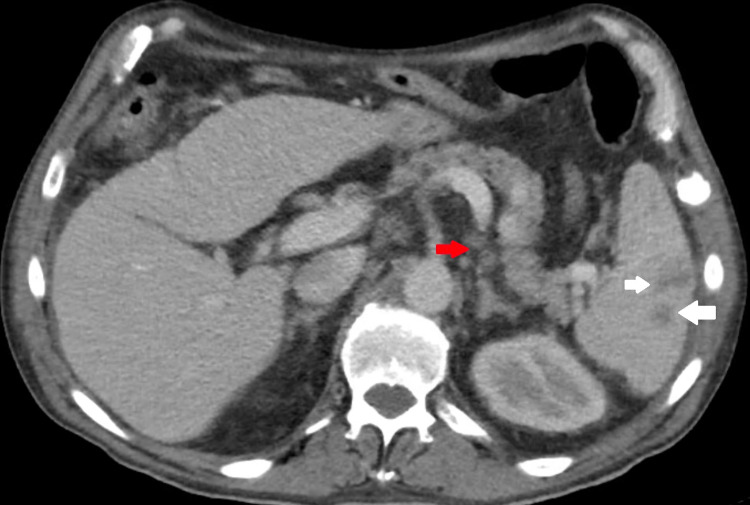
CT abdomen showing the presence of mesenteric and retroperitoneal adenopathy (red arrow) and multiple ill-defined splenic lesions (white arrows) CT: computed tomography

**Figure 3:**
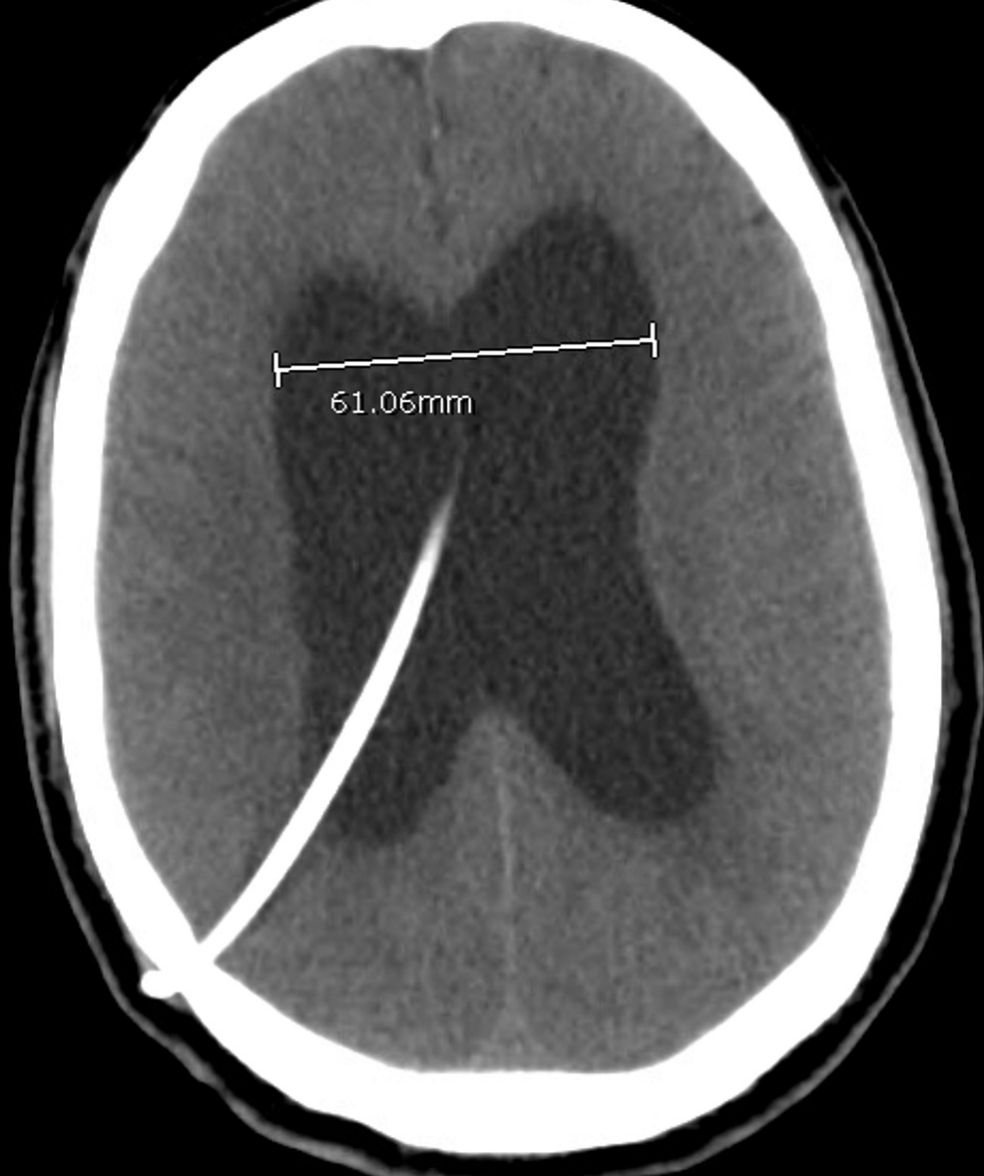
CT brain after the insertion of the VP shunt re-demonstrating ventriculomegaly CT: computed tomography; VP: ventriculoperitoneal

During the current admission, the family reported that the patient slept for more than 22 hours a day. He also had associated daily low-grade fevers, headaches, bilateral lower extremities weakness, ataxia, urinary incontinence, and diffuse mild abdominal pain. The physical exam revealed normal vital signs and bilateral axillary lymphadenopathy. He was somnolent but arousable, oriented to self, had decreased strength of bilateral lower extremities, and had difficulty with his speech. The laboratory investigation revealed a normal complete blood count and comprehensive metabolic panel. HIV and syphilis antibodies were non-reactive. Due to his abdominal pain, a CT scan of the abdomen (Figure 2) was performed, which showed mesenteric and retroperitoneal adenopathy as well as multiple ill-defined splenic lesions; no bowel obstruction or necrosis was visualized, and the findings were suggestive of nonspecific distal enteritis. The brain CT scan (Figure 3) re-demonstrated ventriculomegaly that was out of proportion to the degree of cerebral volume loss (compatible with NPH), stable size of dilated ventricles, and stable gliosis in the right frontal lobe.

**Figure 4:**
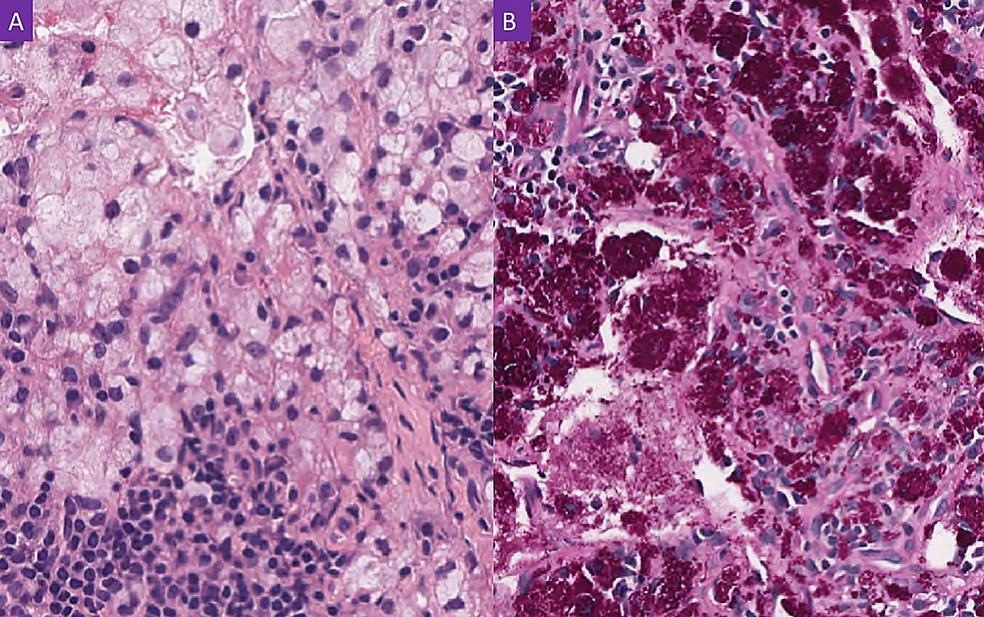
A. Lymph node biopsy with numerous foamy macrophages (H&E X400). B. Special stain shows numerous bacteria in the macrophages (periodic acid-Schiff X400)

The patient later had a seizure and became unresponsive. He was intubated for airway protection. He was started on levetiracetam and empiric IV vancomycin, IV cefepime, and IV acyclovir for meningitis, and the infectious diseases team was consulted. Electroencephalogram (EEG) revealed bifrontal epileptiform discharges. The neurosurgery team adjusted the VP shunt and samples were collected. The CSF analysis showed a white blood cell (WBC) count of 5 cells/µL (20% polymorphonuclear leukocytes, 7% lymphocytes, 73% monocytes), a glucose level of 64 mg/dL, and protein of 31 mg/dL. CSF Gram stain and culture were negative and the antimicrobials were discontinued. Given the patient's history of WD, the infectious diseases team ordered *T. whipplei *PCR from the CSF sample, which returned positive. A retroperitoneal lymph node biopsy showed a diffuse infiltrate of foamy macrophages (Figure 4A) positive for microorganisms by PAS stain (Figure 4B). He was initiated on IV ceftriaxone 2 grams once daily and his condition improved dramatically with the resolution of ventriculomegaly and the removal of the VP shunt. He was continued on IV ceftriaxone for four weeks followed by one year of oral TMP-SMX 160/80 twice daily. His repeat CSF *T. whipplei* PCR at two months was negative. He was followed up regularly at the outpatient clinic, and he subsequently gained weight; he returned to independent living along with a complete resolution of his functional decline and urinary incontinence.

## Discussion

Neurologic involvement in classic WD reportedly ranges from 6 to 63% [1,4,6,7]. The frequency is likely higher considering postmortem and PCR positivity on biopsies or CSF samples of patients without reported neurologic symptoms [1,4]. The isolated CNS WD is less frequent, and previous studies show a prevalence ranging from 4 to 20% [5-8]. In classic WD, the CNS symptoms usually present in the late phase of the disease or as a relapse [6]. The most frequent symptoms are cognitive changes, supranuclear ophthalmoplegia, altered level of consciousness, and psychiatric disorders [3,9]. Meningeal and spinal cord involvement can also occur [5,10]. The risk of neurologic relapse varies based on the type and duration of the treatment [11,12].

We reported a case of a patient with a remote diagnosis of classic WD with incomplete antimicrobial therapy and a more recent diagnosis of NPH with multiple admissions due to functional decline and hypersomnolence. Seneca et al. have described a case of recurrent hydrocephalus managed with ETV similar to our case [13]. These cases show the importance of an early diagnosis and maintaining a high index of suspicion for CNS relapse of WD in patients presenting with neurologic symptoms, after being previously treated for WD, to avoid additional morbidity and mortality.

Our patient had been previously diagnosed with classic WD and treated with TMP-SMX for a few months. The symptoms had improved, but that short course of antibiotics had likely not been enough for the eradication of the bacteria, which often requires treatment for a long period of time. Maiwald et al. have shown evidence of viable bacteria in the CNS in individuals with WD even after a long course of antibiotics [14]. Other authors have reported CNS relapse after a long period of therapy and even during the treatment [5,15]. Another hallmark in our case was the pronounced hypersomnolence of 22 hours in a 24-hour period. Previous reports have shown the occurrence of sleep disturbances and disarrangement of the sleep-wake cycle as the neurologic presentation of WD [16]. The symptoms can be variable, ranging from somnolence to almost complete sleep loss [16,17].

Laboratory exams usually show nonspecific findings such as anemia, leukocytosis, thrombocytosis, and elevation of acute-phase reactants [3]. For neurologic disease, the CSF findings are nonspecific, as in the case of our patient. For the definitive diagnosis, the PCR from CSF samples is a valuable tool and can also be used for routine follow-ups during the treatment [6]. There have been reports of patients without neurologic symptoms and positive CSF PCR [4]. Besides the technical difficulty of a brain biopsy, it demonstrates poor specificity for the PAS staining technique. The association of different techniques and multiple samples from diverse sources, when possible, seems to be the best approach to confirm the diagnosis [1].

The CNS imaging can be variable and can show diffuse cerebral lesions, focal cerebral lesions, cortical or subcortical atrophy, diffuse cerebral edema, hydrocephalus, stroke-like presentation, or normal findings [5,6,18]. According to some reports, a common finding on MRI is high T2 signal intensity with minimal enhancement, either unilateral or bilateral, which are more pronounced on FLAIR sequences [18].

To reduce the risk of relapse, medications with blood-brain barrier penetrance should be used. In the United States, the most common CNS WD regimen used is IV ceftriaxone followed by oral TMP-SMX, while in Europe, doxycycline and hydroxychloroquine seem to be the preferred regimen [19]. The recommended duration of treatment is at least one year of therapy with antibiotics that can be adjusted based on the clinical response. Some authors have also reported the use of interferon γ as adjunctive therapy for specific cases [20].

## Conclusions

It can be challenging to diagnose the neurologic manifestation of WD, a rare presentation of a rare disease. The delay in diagnosis can lead to multiple hospital admissions, permanent sequelae, and fatal outcomes. It is important to consider CNS involvement of WD when a patient with a previous history of classic WD presents with neurologic symptoms.
